# The Nomogram Model Predicting Overall Survival and Guiding Clinical Decision in Patients With Glioblastoma Based on the SEER Database

**DOI:** 10.3389/fonc.2020.01051

**Published:** 2020-06-26

**Authors:** Hongjian Li, Yingya He, Lianfang Huang, Hui Luo, Xiao Zhu

**Affiliations:** ^1^Southern Marine Science and Engineering Guangdong Laboratory (Zhanjiang), The Marine Biomedical Research Institute, Guangdong Medical University, Zhanjiang, China; ^2^Cancer Center, The Affiliated Hospital, Guangdong Medical University, Zhanjiang, China; ^3^School of Foreign Languages, Guangdong Medical University, Dongguan, China

**Keywords:** glioblastoma, nomogram, prognostic factors, predictor, overall survival

## Abstract

**Background:** Patients with glioblastoma have a poor prognosis. We want to develop and validate nomograms for predicting overall survival in patients with glioblastoma.

**Methods:** Data of patients with glioblastoma diagnosed pathologically in the SEER database from 2007 to 2016 were collected by SEER^*^Stat software. After eliminating invalid and missing clinical information, 3,635 patients (total group) were finally identified and randomly divided into the training group (2,183 cases) and the verification group (1,452 cases). Cox proportional risk regression model was used in the training group, the verification group and the total group to analyze the prognostic factors of patients in the training group, and then the nomogram was constructed. C-indexes and calibration curves were used to evaluate the predictive value of nomogram by internal (training group data) and external validation (verification group data).

**Results:** Cox proportional risk regression model in the training group showed that age, year of diagnosis, laterality, radiation, chemotherapy were all influential factors for prognosis of patients with glioblastoma (*P* < 0.05) and were all used to construct nomogram as well. The internal and external validation results of nomogram showed that the C-index of the training group was 0.729 [95% CI was (0.715, 0.743)], and the verification group was 0.734 [95% CI was (0.718, 0.750)]. The calibration curves of both groups showed good consistency.

**Conclusions:** The proposed nomogram resulted in accurate prognostic prediction for patients with glioblastoma.

## Introduction

Glioma is the most common primary central nervous system malignancy in adults, with an annual incidence of 5.26 per 100,000 people (1). In the WHO classification, grade IV glioma is glioblastoma (GBM) (2), which is the highest level (3) in the WHO classification of brain tumors. It is highly malignant (4) and patients have poor prognosis. It is a kind of cancer that is difficult to treat. Conventional treatments for glioblastoma include surgery, alkylation chemotherapy, and radiotherapy. Traditional treatments are often ineffective, not only because glioblastoma is highly invasive, but also because the blood-brain barrier prevents drugs from killing tumor cells completely. Within 2 cm of the primary site, 77% of GBM will recur (5). 72% of the cases will recur in field of radiotherapy (6). These factors are also associated with poor prognosis in patients with GBM.

SEER database (surveillance epidemiology and end results) (https://seer.cancer.gov/) is from the National Cancer Institute (NCI). This database records in detail the demographic information, tumor site and morphology, diagnosis stage, treatment and prognosis of millions of patients with malignant tumors and carcinoma *in situ* in some states since 1973 (7), which provides good data for clinical studies of tumors. Nomogram includes a variety of cancer-related risk factors and presents their impacts on patients' survival in a visual way. It can personally predict the survival rate of patients and it is a common tool for prognosis assessment of cancer patients (8–10). In this study, we extracted the cases of glioblastoma in SEER database from 2007 to 2015, and constructed a nomogram to predict the survival rate of patients and guide clinical prognosis and treatment decisions.

## Patients and Methods

We used SEER^*^Stat (version 8.3.4) to collect 128,554 cases of nervous system tumors diagnosed pathologically. Then Excel 2016 was used to perform data cleaning to eliminate invalid data and select patients with glioblastoma from 2009 to 2015 (Tables 1, 2).

**Table 1:** Cleaning of patient demographic data.

**Variables**	**Before cleaning**	**After cleaning**	
		**Training cohort**	**Verification cohort**
**Age (years)**			
0–49	48,070	394	254
50–54	10,044	271	174
55–59	11,732	309	238
60–64	12,440	338	250
65–69	12,476	314	206
70–74	11,621	267	160
75–79	10,027	170	103
80–84	7,011	93	52
85+	5,133	27	15
**Race**			
Black	8,399	98	82
White	112,750	1,981	1,318
Other	6,872	104	52
Unknown	533		
**Sex**			
Female	56,923	850	602
Male	71,631	1,333	850
**Year of diagnosis**			
1975–2006	73,624		
2007–2009	16,058	744	479
2010–2012	16,341	712	471
2013–2015	17,078	727	502
2016	5,453		
**Type of follow-up expected**			
Active follow-up	126,040		
Autopsy/death certificate only cases	2,423		
SF/Oakland only (originally inactive/now active)	91		
**NHIA (Hispanic, Non-Hisp)**			
Non-Spanish-Hispanic-Latino	114,658	1,927	1,288
Spanish-Hispanic-Latino	13,896	256	164
**Age at diagnosis**			
0–49	48,070	394	254
50–65	36,731	980	705
66+	43,753	809	493
**Type of reporting source**			
Hospital inpatient/outpatient or clinic	122,674		
Others	5,880		
**Insurance**			
Uninsured	1,949	76	48
Medicaid	7,913	239	176
Insured	41,981	1,868	1,228
Unknown	76,711		
**Marital status at diagnosis**			
Single (never married)	33,718	309	208
Unmarried or domestic partner,	68,259	1,500	991
Married (including common law)			
Separated; Divorced; Widowed	22,336	374	253
Unknown	4,241		
**Status**			
Alive	30,554	246	165
Dead	98,000	1,937	1,287

**Table 2:** Cleaning of patient clinical and diagnostic data.

**Variables**	**Before cleaning**	**After cleaning**	
		**Training cohort**	**Verification cohort**
**Site**			
Brain	126,386		
Cranial nerves other nervous system	2,168		
**Behavior**			
Malignant	128,554		
**Site ICD-O-3**			
C710-C719	126,482	2,183	1,452
C700	2,072		
**Histologic type ICD-O-3**			
944	62,703	2,183	1,452
Others	65,851		
**The degree of differentiation**			
Well; moderately; poorly differentiated	18,078	128	87
Undifferentiated; anaplastic	34,705	2,055	1,365
Unknown	75,771		
**Laterality**			
Not a paired site	72,168	259	173
Right - origin of primary	27,278	1,011	649
Left - origin of primary	26,541	913	630
Others	2,567		
**Derived AJCC stage group,7th ed (2010+)**			
Blank(s), NA	128554		
**Derived AJCC T,7th ed (2010+)**			
Blank(s), NA	128554		
**Derived AJCC N,7th ed (2010+)**			
Blank(s), NA	128554		
**Derived AJCC M,7th ed (2010+)**			
Blank(s), NA	128554		
**Primary site surgery**			
0	27,775	344	205
20	15,159	415	265
21	8,838	413	263
30	10,792	461	335
40	12,349	249	162
55	15,678	301	222
10,	131	–	–
22,	96	–	–
90, 99, Blank(s)	37,736		
**Scope region lymph nodes surgery (2003+)**			
Blank(s), unknown or not applicable	128554		
**Other region/distance surgery (2003+)**			
Any combo of sur proc to oth rg, dis lym nd, and/or dis site	8		
None; Blank(s); unknown	128,546		
**Radiation sequence with surgery**			
No radiation and/or cancer-directed surgery	71487	374	223
Radiation (or including surgery)	57067	1,809	1,229
**Reason no cancer-directed surgery**			
Not recommended or not performed	28,904	344	205
Surgery performed	86,124	1,839	1,247
Others; unknown	13,526		
**Radiation**			
Refused or non-beam radiation	3,306	58	34
Beam radiation	73,791	2,125	1,418
Unknown	51,457		
**Chemotherapy**			
No/Unknown	79,585		
Yes	48,69		
**Regional nodes examined (1988+)**			
Unknown; blank(s)	128,554		
**Regional nodes positive (1988+)**			
Unknown; blank(s)	128554		
**Size**			
<=30 mm	12,415	467	316
>30, <=50 mm	19,842	964	643
>50mm	15,804	752	493
Unknown, size not stated, not stated in patient record; not applicable; Blank(s)	80,493		
**Extension**			
All	128,554		
**Cause-specific death classification**			
Alive or dead of other cause	36,099	336	219
Dead (attributable to this cancer dx)	77,697	1847	1233
Dead (missing/unknown COD)	1,232		
N/A not first tumor	13526		
**Other cause of death classification**			
Alive or dead due to cancer	106,693	2,093	1,398
Dead (attributable to causes other than this cancer dx)	7,103	90	54
Dead (missing/unknown COD)	1,232		
N/A not first tumor	13,526		
**Sequence number**			
One primary only	111,771	2,145	1,425
1st of 2 or more primaries	3,257	38	27
Others; unknown	13,526		
**Frist malignant primary indicator**			
Yes	115,778		
No	12,776		
**Total_malig**			
1	112,208		
2	13,925		
3, 4, 5, 6, 7, unknown	2,421		
**Total_begn**			
0	127,149		
1–7, unknown	1,405		

*COD, cause of death; NA, not applicable; ***Total_malig***, total number of in situ/malignant tumors for patient; ***Total_begn***, Total number of benign/borderline tumors for patient. In ***Site ICD-O-3***, code C710-C719 mean “brain or cranial nerves other nervous system,” code C700 means “cranial nerves other nervous system.” In ***Histologic Type ICD-O-3***, code 944 means glioblastoma. In ***Primary site surgery***, code 0 means “none/no surgery of primary site/autopsy only”; code 10 means “tumor destruction, NOS (not otherwise specified)”; code 20 means “local excision of tumor, lesion, or mass, excisional biopsy”; code 21 means “subtotal resection of tumor, lesion or mass in brain”, code 22 means “resection of tumor in spinal cord or nerve”; code 30 means “radical, total, gross resection of tumor, lesion or mass in brain”; code 40 means “partial resection of lobe of brain, when the surgery cannot be coded as 20-30”; code 55 means “gross total resection of lobe of brain (lobectomy)”; code 90 means “surgery, NOS”; 99 means “unknown if surgery performed; death certificate only”*.

**Figure 1:**
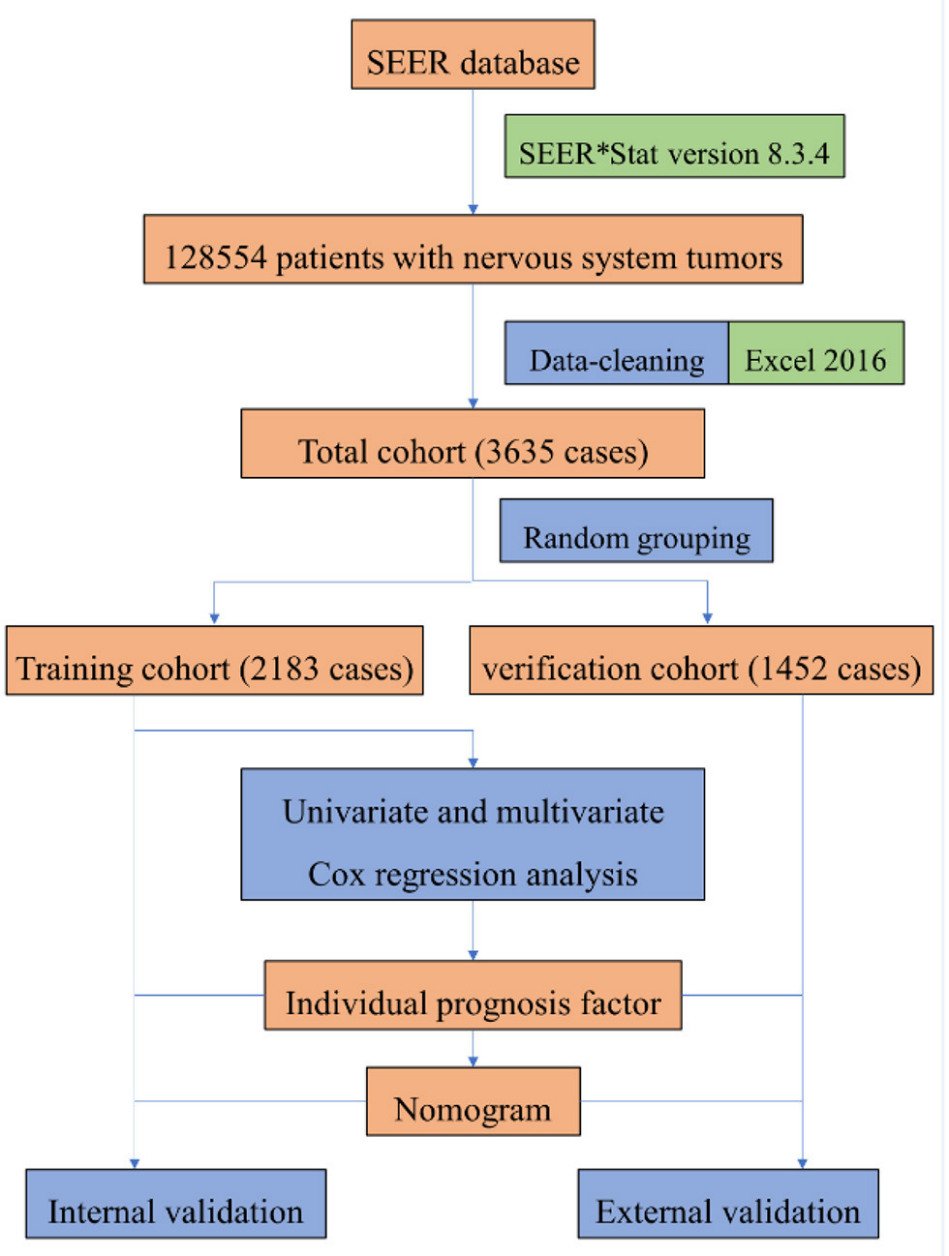
The procedure of building a nomogram for patients with glioblastoma. Firstly, we derived patients with nervous system tumors from the SEER database, then selected patients with glioblastoma, and eliminated the invalid data. Secondly, univariate and multivariate Cox regression analysis were performed to obtain individual variables affecting prognosis. Thirdly, individual variables were used to construct nomogram that predicted the prognosis of patients, then internal and external validation were performed.

The data set partitioning function [createDataPartition] in the “caret” package of R version 3.5.3 was used for random grouping, so the cleaned data of patients were randomly divided into training group and verification group. In the training group, “survival” package was used for univariate and multivariate Cox proportional risk regression model analysis to screen prognostic factors, and “rms” package was used to construct nomogram. In nomogram's external validation, we calculated the total points of each patient based on the constructed nomogram in the verification group and Cox regression was performed with the total points as a factor. We calculated the C-index and drew calibration curve by Bootstrap method (resampling number B=100) in the training group and the verification group, respectively, for internal and external validation. The higher the C-index is, the more accurate the prognosis is (11). In the calibration curve, if the predicted value is equal to the actual observed value, the curve will be infinitely close to the ideal 45° slant (12). Risk score was constructed, and ROC (receiver operating characteristic) curve was derived to evaluate the predicting value. Both the C- index and area under the ROC curve (AUC) can be used to evaluate the discrimination between the real value and the predicted value of the model (12). Our study procedure is shown in Figure 1.

## Results

### Demographics and Clinicopathologic Characteristics of Patients

This study included the following characteristics into the analysis: age, race, sex, year of diagnosis, degree of differentiation, laterality, primary site surgery, radiation sequence with surgery, reason no cancer-directed surgery, radiotherapy, chemotherapy, tumor size, cause-specific death classification, other cause of death classification, sequence number, NHIA (Hispanic, Non-Hisp), age at diagnosis, insurance, marital status at diagnosis (Table 3).

**Table 3:** Univariate and multivariate analyses of prognostic parameter in glioblastoma using the Cox regression model.

**Variables**	**Univariate cox**			**Multivariate cox**		
	**Hazard ratio**	**95% CI**	***p*–value**	**Hazard ratio**	**95% CI**	***p*–value**
**Age**						
<50	–	–	–	–	–	–
50–54	1.70116	1.437–2.014	6.88 × 10^−10^	1.154	0.8227–1.6185	0.40702
55–59	1.65626	1.405–1.953	1.99 × 10^−9^	1.204	0.8605–1.6858	0.27823
60–64	1.80213	1.536–2.115	5.34 × 10^−13^	1.362	0.9747–1.9046	0.07032
65–69	2.06971	1.759–2.435	<2 × 10^−16^	1.734	1.4534–2.0699	1.02 × 10^−9^
70–74	2.55537	2.157–3.028	<2 × 10^−16^	1.961	1.6460–2.3371	4.95 × 10^−14^
75–79	3.699	3.054–4.480	<2 × 10^−16^	2.678	2.2011–3.2585	<2 × 10^−16^
80–84	5.42584	4.276–6.885	<2 × 10^−16^	3.666	2.8617–4.6966	<2 × 10^−16^
≥85	6.58357	4.428–9.789	<2 × 10^−16^	3.509	2.3364–5.2689	1.44 × 10^−9^
**Race**						
Black	–	–	–			
White	0.98572	0.7993–1.216	0.8930			
Others	0.74720	0.5553–1.005	0.0543			
**Sex**						
Female	–	–	–			
Male	1.02670	0.9367–1.125	0.573			
**Year of diagnosis**						
2007–2009	–	–	–	–	–	–
2010–2012	0.91054	0.8192–1.8192	0.0823	1.114	0.9850–1.2609	0.08540
2013–2015	0.88406	0.7900–0.9893	0.0317	1.524	1.3264–1.7514	2.79 × 10^−9^
**The degree of differentiation**						
Well; moderately; poorly differentiated	–	–	–			
Undifferentiated; anaplastic	1.14822	0.9486 −1.39	0.156			
**Laterality**						
Not a paired site	–	–	–	–	–	–
Right	0.66153	0.5749–0.7612	7.80 × 10^−9^	0.8288	0.7185–0.9560	0.00997
Left	0.62286	0.5405–0.7178	6.16 × 10^−11^	0.7715	0.6678–0.8914	0.00043
**Surg prim site (1998+)**						
0	–	–	–			
20	0.56257	0.4850–0.6526	3.10 × 10^−14^			
21	0.60341	0.5191–0.7014	4.64 × 10^−11^			
30	0.46608	0.4020–0.5404	<2 × 10^−16^			
40	0.63234	0.5356–0.7465	6.27 × 10^−8^			
55	0.51739	0.4407–0.6075	8.56 × 10^−16^			
**Radiation sequence with surgery**						
No radiation and/or cancer–directed surgery						
Radiation (or including surgery)	0.51393	0.4583–0.5763	<2 × 10^−16^			
**Reason no cancer–directed surgery**						
Not recommended/not performed	–	–	–			
Surgery performed	0.54541	0.4846–0.6138	<2 × 10^−16^			
**Radiation**						
Refused or non–beam radiation	–	–	–			
Beam radiation	0.3623	0.2775–0.4729	8.34 × 10^−14^	0.5939	0.3981–0.8859	0.01066
**Chemotherapy**						
No/unknown	–	–	–			
Yes	0.41750	0.3672–0.4747	<2 × 10^−16^	0.5554	0.4829–0.6388	<2 × 10^−16^
**Size (mm)**						
≤30	–	–	–			
>30, ≤50	1.007	0.8952–1.133	0.905			
>50	1.014	0.8961–1.147	0.826			
**Cause–specific death classification**						
Alive or dead of other cause	–	–	–			
Dead (attributable to this cancer dx)	7.3289	5.914–9.083	<2 × 10^−16^			
**Other cause of death classification**						
Alive or dead due to cancer	–	–	–			
Dead (attributable to causes other than this cancer dx)	7.3289	5.914–9.083	<2 × 10^−16^			
**Sequence number**						
One primary only	–	–	–			
1st of 2 or more primaries	0.7093	0.4915–1.024	0.0665			
**NHIA (Hispanic, Non–Hisp)**						
Non–Spanish–Hispanic–Latino	–	–	–			
Spanish–Hispanic–Latino	0.8742	0.7593–1.006	0.0615			
**Age at diagnosis**						
0–49	–	–	–			
50–65	1.74	1.525–1.984	<2 × 10^−16^			
≥66	2.713	2.370–3.104	<2 × 10^−16^			
**Insurance**						
Uninsured	–	–	–			
Medicaid	0.9716	0.7397–1.276	0.836			
Insured	1.0055	0.7902–1.279	0.965			
**Marital status at diagnosis**						
Single	–	–	–			
Married or partner	1.19626	1.045–1.369	0.00916			
Separated, divorced or widowed	1.44977	1.232–1.706	7.86 × 10^−6^			

*Meaningless variables in statistical results are not listed in this table*.

### Independent Prognostic Factors in the Training Group

**Figure 2:**
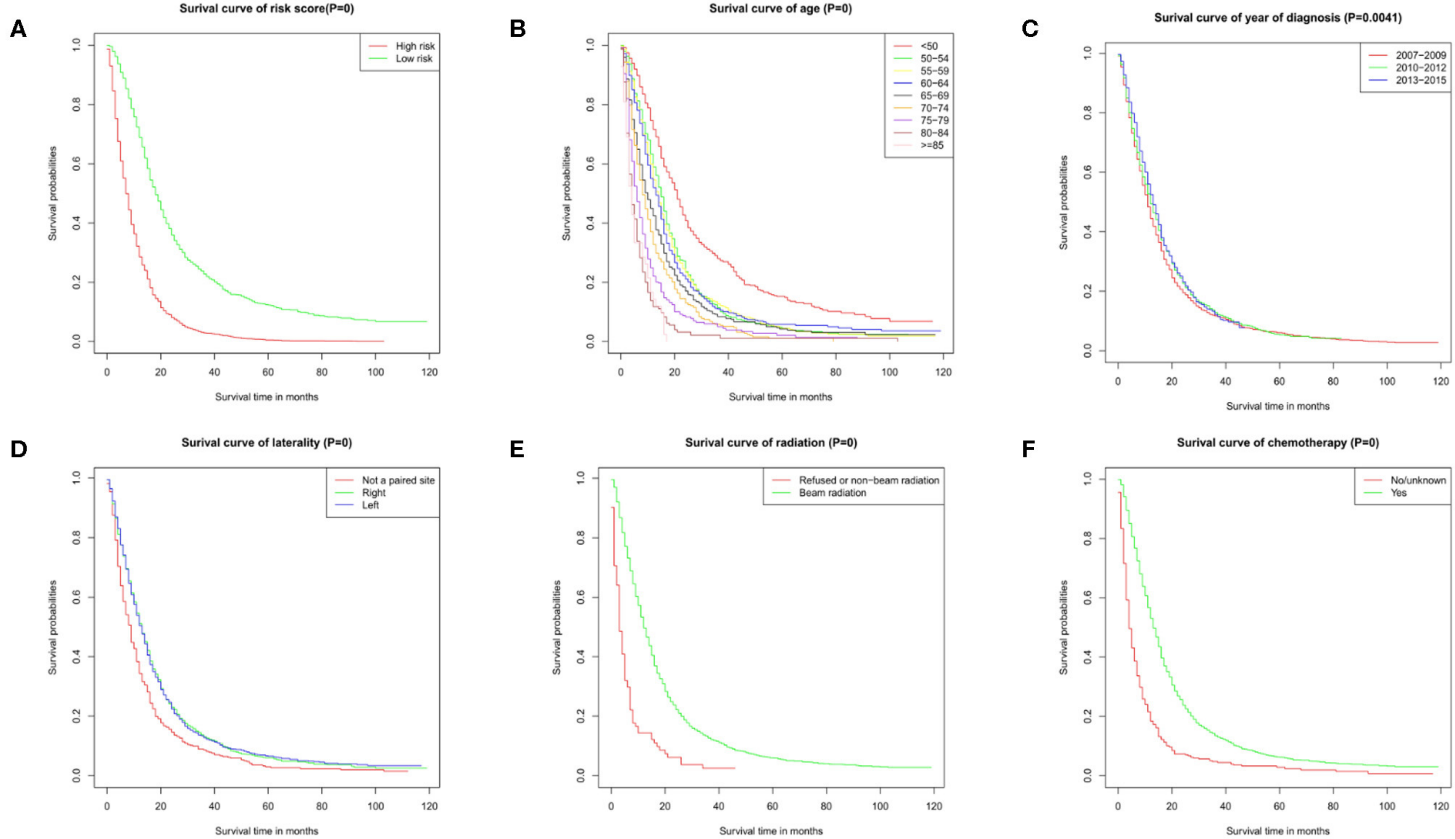
Survival curves. Survival curve of **(A)** risk score, **(B)** age, **(C)** year of diagnosis, **(D)** laterality, **(E)** radiation, and **(F)** chemotherapy. These graphs show the impact of each subtype on survival. *P* = 0 means *P* < 0.001.

The results of the univariate analysis have been listed in Table 3. Multivariate Cox analyses demonstrated that age, year of diagnosis, laterality, radiotherapy, chemotherapy were independent risk factors for overall survival (OS) (Figure 2A).

### Prognostic Nomogram for OS

**Figure 3:**
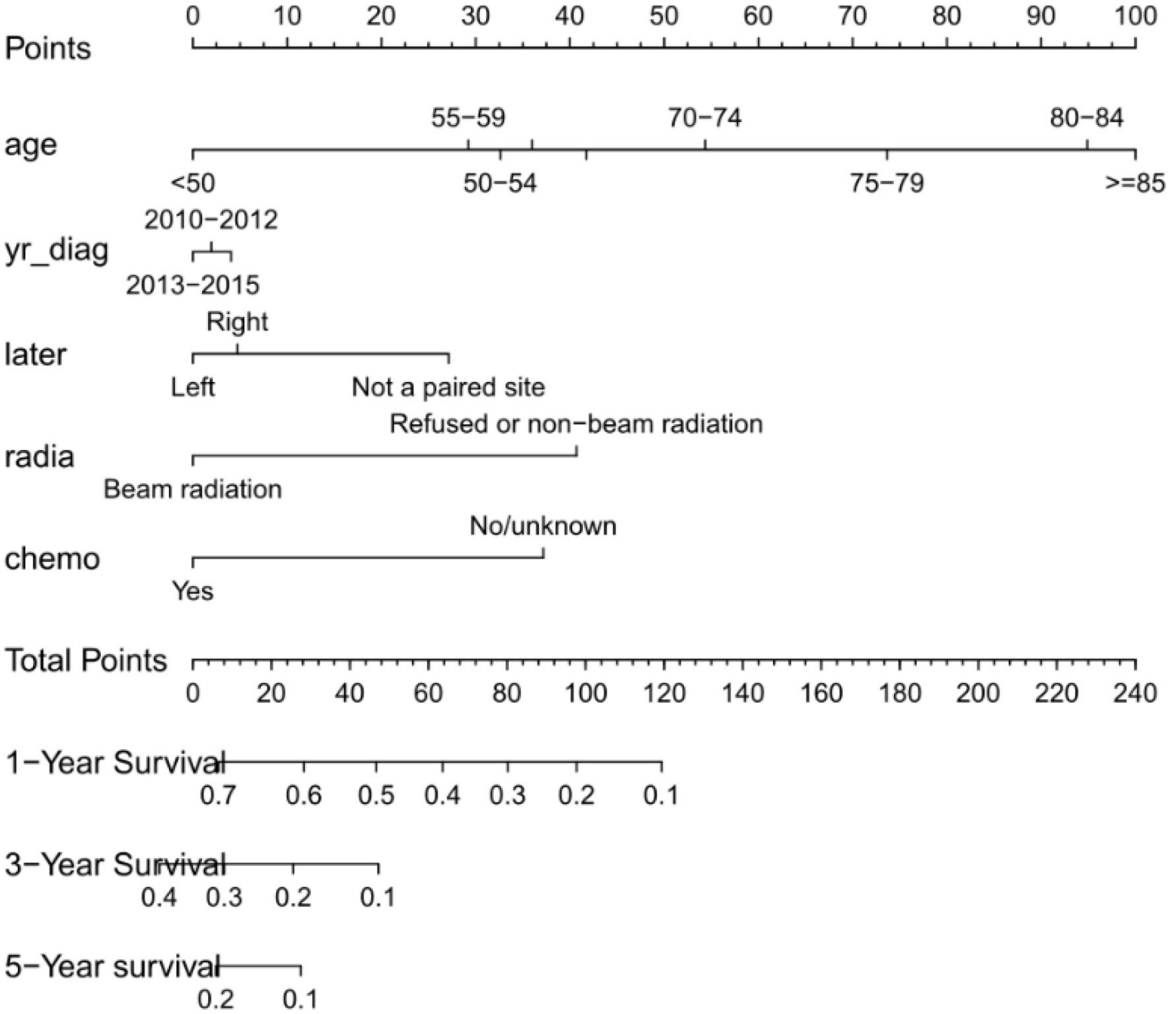
Nomogram predicting patients' 1 -, 3 -, and 5-years over survival with glioblastoma. The specific subtypes of each variables are projected to the point scale to obtain a value. The higher the value, the worse the prognosis of patients. The total value can be obtained by summing up the values of each variables. The 1-year, 3-years, and 5-years over survival of the patient can be obtained by downward projection of the total value in the total point scale. yr_diag, year of diagnosis; later, laterality; radia, radiation; chemo, chemotherapy.

**Figure 4:**
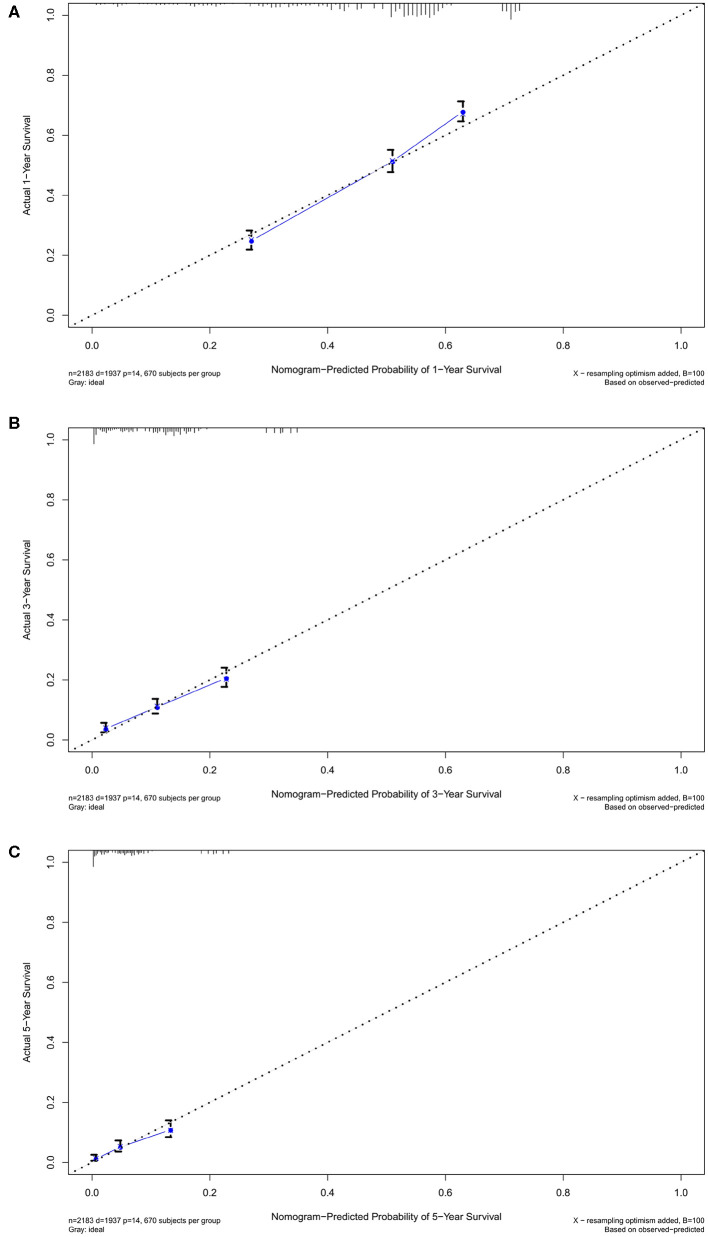
The calibration curves in training cohort. The calibration curves of the nomogram predicting **(A)** 1-year, **(B)** 3-years, and **(C)** 5-years OS.

**Figure 5:**
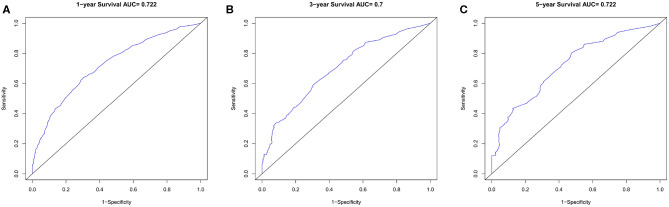
The ROC curves in training cohort. **(A)** 1-year, **(B)** 3-years, **(C)** 5-years survival ROC curves.

Nomogram was built by the “rms” package in R version 3.5.3 based on the results of multivariate analysis (Figure 3). The *rcorrp.cens* package in *Hmisc* was used to calculate the C-index for measuring the performance of the nomogram. The C-index for OS prediction was 0.729 (95% CI, 0.715–0.743). Calibration curves of 1-, 3-, or 5-years survival rates show good agreement between nomogram predictions and actual observations (Figures 4A–C). In training group, 1-, 3-, and 5-years survival AUCs were 0.722, 0.700, and 0.722, respectively (Figures 5A–C). Figures 2B–F showed the OS of the patients with GBM, and the survival curve declined sharply in the first 20 months.

### Validation of Predictive Accuracy of the Nomogram for OS

**Figure 6:**
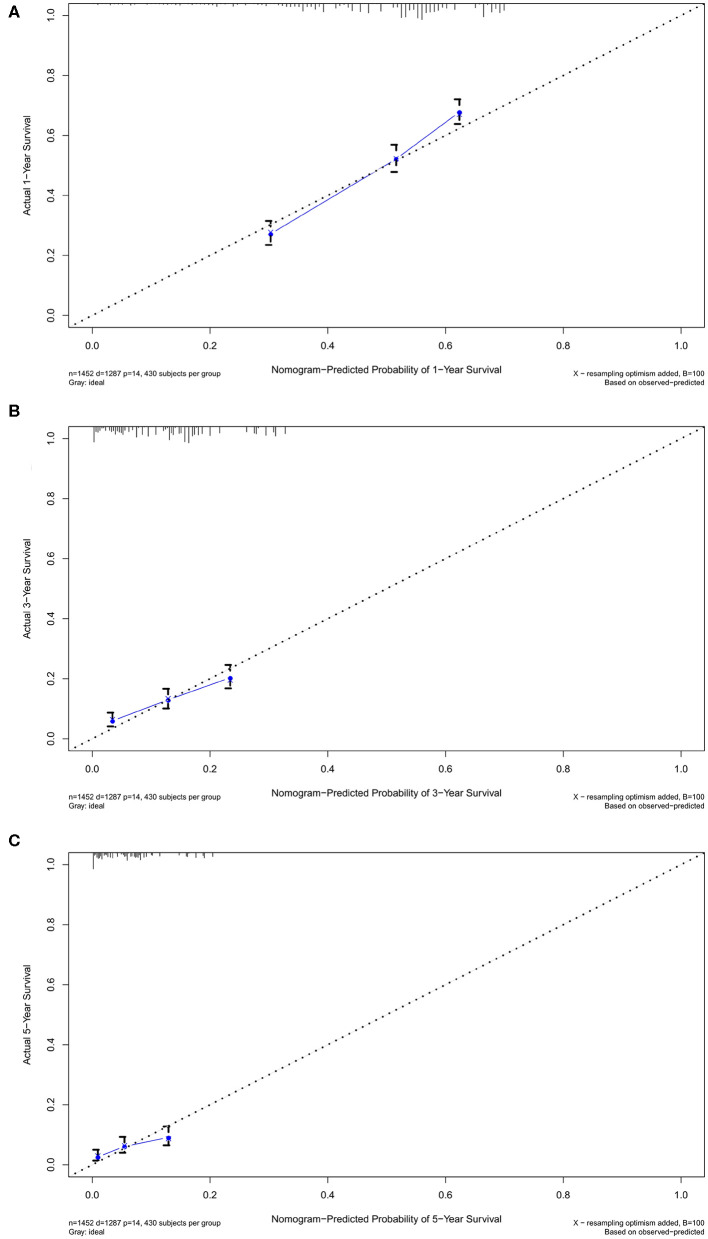
The calibration curves in verification cohort. The calibration curves of the nomogram predicting **(A)** 1-year, **(B)** 3-years, and **(C)** 5-years OS.

**Figure 7:**
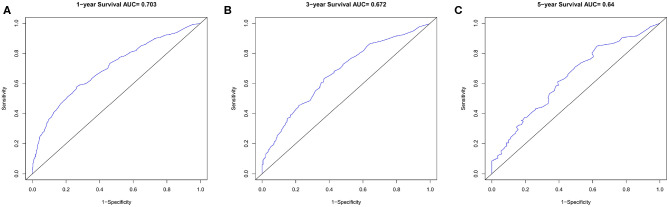
The ROC curves in verification cohort. **(A)** 1-year, **(B)** 3-years, **(C)** 5-years survival ROC curves.

In the verification group, the C-index of the nomogram for predicting OS was 0.734 (95% CI, 0.718–0.750), and calibration curves showed good agreement between nomogram-predicted probability and actual observations of 1-, 3-, or 5-years survival (Figures 6A–C). In verification group, 1-, 3-, and 5-years survival AUCs were 0.703, 0.672, and 0.640, respectively (Figures 7A–C).

## Discussion

Accurate and effective prognosis assessment is of clinical significance for individualized treatment and follow-up treatment of patients with GBM. GBM is usually diagnosed in late stage (13) by MRI with poor prognosis, therefore, a complete prognostic scoring system is essential. Nomogram is a statistical tool that integrates a variety of prognostic risk factors and visualizes the overall impact of these risk factors on survival in each patient (14) to help clinicians develop intervention plan. Compared with other rating systems (such as AJCC 8th edition TNM staging system and the Nathan staging system), nomogram is more convenient and accurate, with a higher C-index, showing better predictive value (15, 16). Many scholars have produced nomograms for some tumors, such as intrahepatic cholangiocarcinoma (17), invasive pulmonary adenocarcinoma (18), colorectal cancer (19), hepatocellular carcinoma with pulmonary metastasis (20), etc. SEER collects 450,000 cancer cases with high-quality information each year, and adjusts the collection of cancer staging information according to changes in cancer staging systems, such as AJCC (7). So that it provides a good data basis for establishing of nomogram.

To construct the nomogram, independent predictors of OS in patients with GBM should be determined first. Univariate and multivariate Cox proportional risk regression models were used to determine independent prognostic factors for OS. Multivariate Cox regression proportional analysis showed that age, year of diagnosis, laterality, radiation, chemotherapy were independent prognostic factors for OS in patients with GBM.

It is an acceptable view that the prognosis of cancer patients is worse with aging. Ladomersky et al. analyzed several databases (SEER, GTEx, and 10 k Immunomes) and found that the death rate of patients with GBM over 65 years old was more than seven times higher than that of patients under 65 years old, thus the prognosis of patients with GBM over 65 years old was much worse than that of patients with GBM under 65 years old (21). In our study, the results of multivariate analysis further showed that, for patients aged at least 65 years old, the older they were, the higher the HR (hazard ratio) was and the worse the prognosis was. That was similar to the results of Bartek's SEER based study, which showed a 0.8% increased risk of death from glioblastoma with each additional year of age at diagnosis (CI 1.008–1.008, *p* < 0.001) (22). Elderly patients are more likely to develop other high-risk complications (23–26). In addition, aging may promote the initiation or growth of GBM cells by suppressing the immune system, and may reduce the effectiveness of immunotherapy for patients with glioblastoma (21). All those will reduce the prognostic survival rate of patients with GBM.

For newly diagnosed glioblastoma, the most important treatment is the resection of the contrast-enhanced region on imaging, followed by radiotherapy and chemotherapy (27, 28). The results of our multivariate analysis showed that both beam radiotherapy and chemotherapy can immensely reduce the risk of patients with glioblastoma and improve the survival rate. Yaprak et al. (29) and Liao et al. (30) proved the feasibility of radiotherapy for glioblastoma. In our study, the prognosis of patients with beam radiation therapy was better than with non-beam radiation therapy (including radioactive implants, brachytherapy, radioisotopes, etc.) or without radiotherapy. In addition, the results of our study indicate that patients with chemotherapy have a significantly higher survival rate than those without chemotherapy. This may be an important role played by temozolomide, the first-line chemotherapy drug for glioblastoma. Temozolomide's introduction significantly improved the prognosis of patients with glioblastoma. The Stupp protocol, proposed in 2005, is the standard of treatment for glioblastoma. It includes radiotherapy, concomitant and adjuvant chemotherapy with temozolomide (28). This kind of treatment significantly improved survival. Perry et al. (31) randomly divided 562 newly diagnosed glioblastoma patients over the age of 65 into two groups, one receiving only short-term radiation therapy and the other receiving short-term radiation therapy plus adjuvant temozolomide. The results showed that the median overall survival of patients who received radiotherapy with temozolomide was longer than those who received chemotherapy alone (5.3 months vs. 3.9 months; *P* < 0.001).

The impact of tumor laterality on the prognosis of patients has not been fully investigated so far. Daniel et al. retrospectively analyzed 235 cases of patients with glioblastoma on surgical outcome, which were grouped for left—and right—sided GBM (32). The results showed that KPS (Karnofsky Performance Status) decreased significantly and PFS (progression-free survival) was shorter in patients with left hemisphere tumors, but their OS was not significantly different from those in patients with right hemisphere tumors. In our study, patients whose tumor originated at paired site (e.g., left or right hemisphere) had a better prognosis than those whose tumor originated at non-paired site. And patients whose tumors originated at the left had a lower risk than those whose tumors that originated at the right. Tumor laterality may be a noteworthy prognostic factor because different regions of the brain perform different functions, and neurologists may use conservative therapy to preserve some of the patient's functions for need.

Based on the above results, we propose the following suggestions for the treatment of patients with glioblastoma. Pay attention to the age of the patient, especially those aged 65 or older, because elderly people have a worse prognosis. Radiotherapy (especially beam radiotherapy) and chemotherapy should be used, as they can significantly improve the prognosis of patients with glioblastoma. Increase attention to tumor laterality, it may also be a potential prognostic factor.

However, our study has some limitations. Firstly, the SEER database does not include information about tumor markers, such as MGMT (O (6)-methylguanine-DNA methyltransferase) promoter methylation (33) and IDH (isocitrate dehydrogenase) mutation (34), and the two markers have strong prognostic value in patients with glioblastoma. Secondly, the SEER database lacks records of neurological interventions, so this study was not discussed. Thirdly, the study objects of SEER, the American clinical database of tumors, are predominantly white people and black people but few Asians. That partly limited the application of our nomogram in Asian patients. Fourthly, as a retrospective study, both the training group and the verification group may be affected by selection bias.

## Conclusion

In conclusion, nomogram integrates easily available factors and serves as an easy-to-use tool to assist patients with glioblastoma in risk assessment and clinical decision-making. The nomogram proposed in this study may objectively and accurately predict 1-, 3, and 5-year OS of patients with glioblastoma. Additional studies are needed to determine whether it is appropriate for the other patient group.

## Data Availability Statement

Publicly available datasets were analyzed in this study. This data can be found here: https://seer.cancer.gov/.

## Author Contributions

HLi and XZ performed the statistical analyses. XZ checked the statistical accuracy as an expert in statistics. HLi performed the literature search and wrote the first draft of the manuscript. HLi, LH, YH, HLu, and XZ revised and edited the final version of the manuscript. All authors read and approved the final manuscript.

## Conflict of Interest

The authors declare that the research was conducted in the absence of any commercial or financial relationships that could be construed as a potential conflict of interest.
